# Three-dimensional design custom-made uncemented stem for revision of cemented distal femoral endoprosthesis due to aseptic loosening

**DOI:** 10.1186/s13018-023-03994-0

**Published:** 2023-07-17

**Authors:** Zhuangzhuang Li, Minxun Lu, Yong Zhou, Li Min, Chongqi Tu

**Affiliations:** 1 Department of Orthopedics, Orthopaedic Research Institute, West China Hospital, Sichuan University, No. 37 Guoxue Road, Chengdu, 610041 Sichuan People’s Republic of China; 2 Model Worker and Craftsman Talent Innovation Workshop of Sichuan Province, West China Hospital, Sichuan University, Chengdu, People’s Republic of China

**Keywords:** Distal femoral replacement, Aseptic loosening, Revision, Uncemented stem, 3D design

## Abstract

**Background:**

Revision of cemented distal femoral replacement (DFR) due to aseptic loosening is challenging because of the resultant femoral bone loss. This paper aims to examine the outcomes of three-dimensional (3D) design custom-made uncemented stems for revision.

**Methods:**

Between January 2014 and December 2020, 17 patients received 3D design uncemented stems for revision of loosed cemented DFR. The femoral bone loss was classified into four Grades, and four types of uncemented stems were designed correspondingly. The revision stems were custom-made for each patient by measuring the diameter of the medullary cavity and the anterior curvature of the femur.

**Results:**

The patient counts with their corresponding Grades of femoral bone loss were as follows: Grade I, 8 patients; Grade II, 5 patients; Grade III, 3 patients; and Grade IV, 1 patient. During the mean follow-up of 80 months, no revision failure was detected. The postoperative radiographic showed that the stem matched the femoral anterior curvature well. The femoral bone defect was completely filled by the 3D design stem in 10 of the 17 cases postoperatively. In the remaining cases, the persistent peri-stem defect was filled or partially restored during the follow-up.

**Conclusion:**

3D design custom-made uncemented stem created precise, stable, and durable fixation and provided satisfactory clinical outcomes, which seems to be a viable method for cemented DFR revision.

## Background

Limb salvage surgery is currently the standard treatment for distal femoral bone tumors, and distal femoral replacement (DFR) has become the preferred reconstruction option after tumor resection [1, 2]. However, in long-term follow-up, replacement failure is common for most of the patients, and therefore revision surgery is frequently performed [3]. Henderson et al. classified the failure into five types: soft tissue failure, aseptic loosening, structure failure, infection, and tumor progression [4]. Among them, aseptic loosening is the most common reason for replacement failure, especially for cemented DFR [5–8].

Progressive bone loss and periprosthetic osteolysis after aseptic loosening often lead to poor bone quality and resultant insufficient bone stock, which makes revision surgery challenging [9, 10]. Total femur replacement is a relatively simple procedure for revision following DFR failure [11], while the drawback of sacrificing the innocent hip joint restricts its wide application. In addition, removing the loosed femoral cemented stem and fixing the revision endoprosthesis to the residual femur have been described, and the commonly used fixation techniques include compress osteointegration [3], cemented stem [9], and uncemented stem [12]. However, the optimal method remains controversial. Recently, the utilization of an uncemented stem has gained popularity due to its remarkable advantages, such as the potential to achieve biologic and possibly permanent fixation [13].

Previously, we have presented three-dimensional (3D) design custom-made uncemented stem for revision of aseptic loosening of cemented DFR, which creates precise and stable fixation with a mean follow-up of 24 months [12]. In this paper, our aim is to extend to examine the outcomes of 3D design custom-made uncemented stems further.

## Materials and methods

### Patients

This retrospective study was performed after obtaining approval from the ethics committee. Between January 2014 and December 2020, 17 patients received 3D design custom-made uncemented stems for revision of loosed cemented DFR. All patients in this study met the following criteria: (1) Primary bone tumor diagnosed at the distal femur; (2) Primary DFR with cemented endoprosthesis after tumor resection; (3) Revision surgery due to aseptic loosening of the femoral stem; and (4) With a minimum follow-up of 2 years after revision surgery.

Among the 17 patients, there were 7 females and 10 males, with a mean age of 37 years at the time of revision surgery. Oncologic diagnoses included osteosarcoma (10 cases), giant cell tumors of bone (five cases), and chondrosarcomas (two cases). Before revision surgery, all patients underwent detailed radiographic examinations of the affected limb, including X-ray, 3D computed tomography (CT), and magnetic resonance imaging (MRI). In addition, chest CT was examined to exclude pulmonary metastasis, and full-body bone isotope scans were conducted if necessary. Preoperative blood routine, erythrocyte sedimentation rate, and C reactive protein biochemical routine were conducted to rule out infection. For all patients, the pain level was evaluated using the Visual analog scale (VAS, 0–10) method, with 0 indicating no pain and 10 indicating unbearable pain. The limb function was assessed by the Musculoskeletal Tumor Society (MSTS) scoring system. The patient’s demographic data and clinical characteristics are shown in Table 1. The interval between primary reconstruction and revision surgery and the endoprosthesis type of initial DFR were collected.

**Table 1:** Patients’ characteristics and the initial cemented distal femur replacement information

Case	Age	Gender	Side	Pathological diagnosis	Initial endoprosthesis	Survival time of initial endoprosthesis (years)	Grade of bone loss
1	21	M	L	OS	FHE	2	I
2	35	M	R	GCT	FHE	4	II
3	27	M	L	OS	RHE	5	I
4	26	F	L	CS	RHE	6	I
5	49	M	R	OS	RHE	8	II
6	22	F	R	GCT	RHE	5	I
7	32	M	R	OS	RHE	3	II
8	35	M	L	OS	RHE	17	II
9	28	M	R	OS	RHE	12	III
10	44	F	R	GCT	RHE	6	II
11	28	M	R	OS	FHE	15	I
12	67	M	L	OS	RHE	6	III
13	25	M	L	OS	RHE	7	I
14	56	M	L	GCT	RHE	10	I
15	25	F	L	OS	RHE	7	I
16	70	M	R	GCT	FHE	7	III
17	43	F	R	CS	RHE	9	IV

*M* male, *F* female, *L* left, *R* right, *OS* osteosarcoma, *GCT* giant cell tumor, *CS* Chondrosarcoma, *FHE* fixed hinge endoprosthesis, *RHE* rotating hinge endoprosthesis

### Revision planning and stem design

**Fig. 1:**
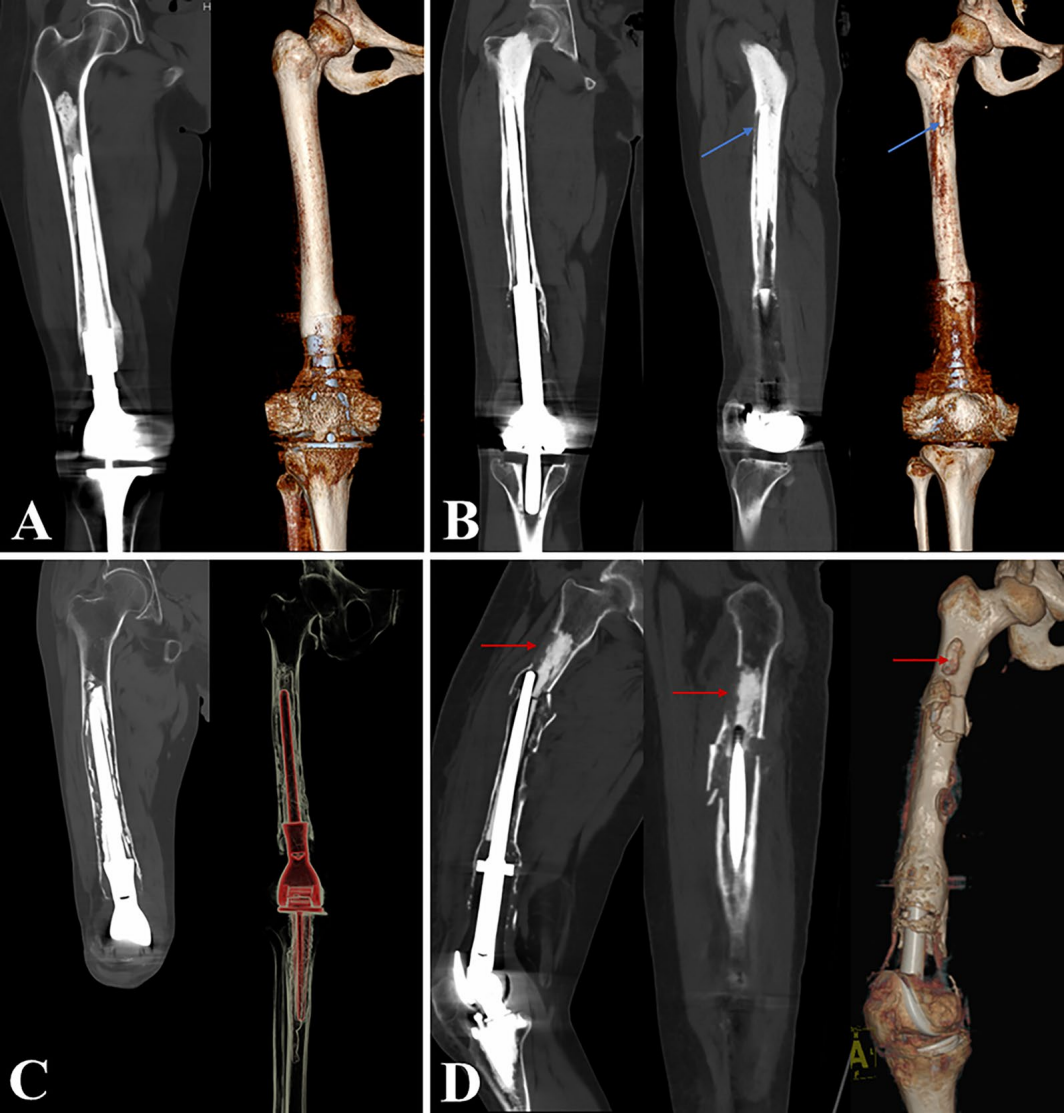
4 Grades of femoral bone loss after aseptic loosening: Grade I (minimal bone loss) (**A**); Grade II (moderate bone loss) (**B**); Grade III (major bone loss) (**C**); Grade IV (severe bone loss) (**D**)

The revision surgery was planned on preoperative radiographic results, and the revision stem was custom-made for each patient. This procedure included three main steps: evaluation and classification of femoral bone loss after aseptic loosening, stem design, and fabrication. Firstly, X-ray and CT scans were evaluated by two independent orthopaedic oncologists, and the femoral bone loss was classified into 4 grades (Fig. 1). Grade I (minimal bone loss): circumferential radiolucency with mild cortical thinning or osteolysis, and the femoral medullary canal was intact. Grade II (moderate bone loss): circumferential radiolucency with mild cortical thinning or osteolysis, while the femoral stem penetrated the cortical bone. Grade III (major bone loss): extensive cortical thinning with osteolysis, with or without the femoral stem penetrating the cortical bone; while the medullary canal at the lesser trochanter was intact. Grade IV (severe bone loss): deficiency of most of the femoral bone with extreme cortical thinning or osteolysis, and the medullary canal at the lesser trochanter was nonsupportive for the traditional stem. Meanwhile, based on the radiographic results, the endoprosthetic components were evaluated. If the initial DFR was not modular, or severe wearing of the joint liner was detected, the endoprosthetic components were replaced along with the femoral stem. Otherwise, only the femoral stem required replacement (Table 2).

**Table 2:** Details of revision and follow-up of 17 patients

Case	Revision requirement	Revision stem type	Revision stem length (mm)	Antecurvature radian of the revision stem	Follow-up (months)	VAS (Pre/Pos)	MSTS (Pre/Pos)	Complications
1	Stem	Short stem	110	4	126	4/1	17/28	
2	Stem	Long stem	150	3	122	5/2	18/27	
3	Stem and components	Short stem	110	4	120	4/0	13/26	
4	Stem and components	Short stem	110	5	120	4/1	15/27	
5	Stem and components	Long stem	180	3	114	3/0	15/24	
6	Stem and components	Short stem	110	3	108	5/1	16/26	
7	Stem	Long stem	170	3	108	5/2	18/25	
8	Stem	Long stem	160	4	95	7/3	19/27	DWH
9	Stem	Ultra-long stem	180	3	82	6/2	16/23	
10	Stem and components	Long stem	170	4	78	4/0	15/25	
11	Stem and components	Short stem	110	5	60	5/1	18/26	
12	Stem and components	Ultra-long stem	190	4	55	5/2	19/28	
13	Stem and components	Short stem	100	3	43	4/1	13/26	DWH
14	Stem and components	Short stem	100	5	36	5/0	14/22	
15	Stem and components	Short stem	100	5	29	3/0	16/24	
16	Stem and components	Ultra-long stem	180	4	25	3/2	17/23	
17	Stem and components	Intra-neck curved stem	–	–	42	6/1	13/27	

*VAS* Visual analog scale, *Pre* preoperative, *Pos* postoperative, *MSTS* the Musculoskeletal Tumor Society (MSTS) scoring system, *DWH* delayed wound healing

**Fig. 2:**
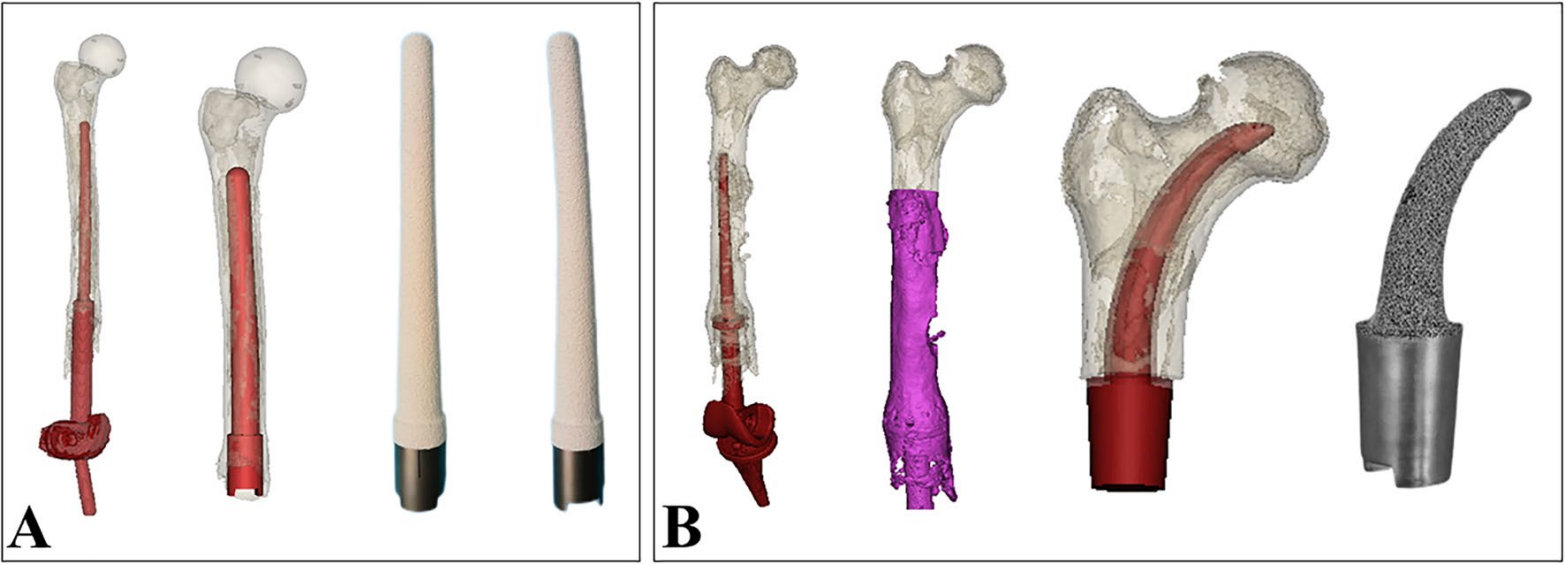
Revision planning and 3D design of **A** intra-diaphysis curved uncemented stem and **B** intra-neck curved uncemented stem

**Fig. 3:**
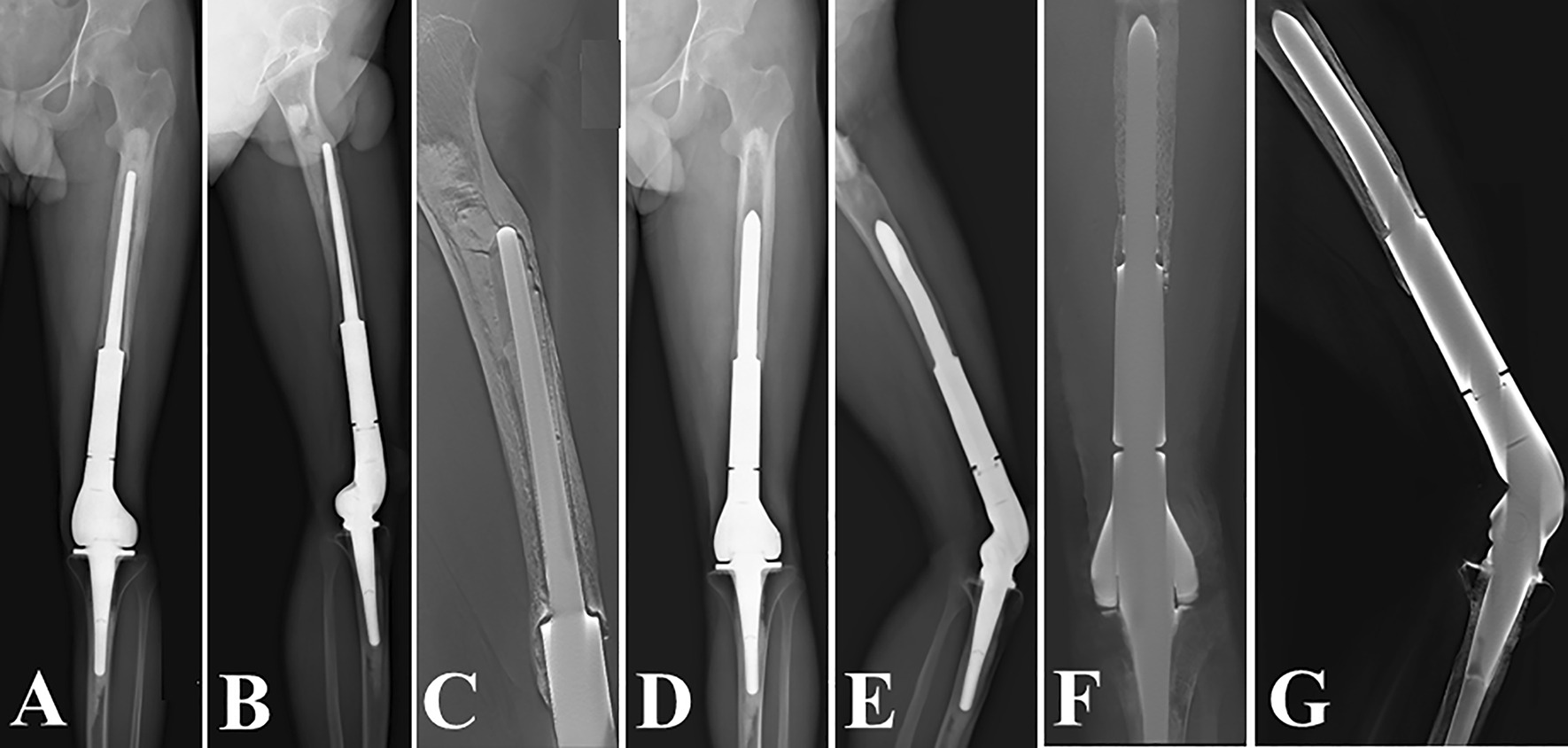
Revision of a Grade I bone loss case with “Short uncemented stem”. **A**–**C** preoperative radiographs; the anteroposterior and lateral radiographs (**D**, **E**), and the digital tomosynthesis images (**F**, **G**) 1 year after revision surgery

**Fig. 4:**
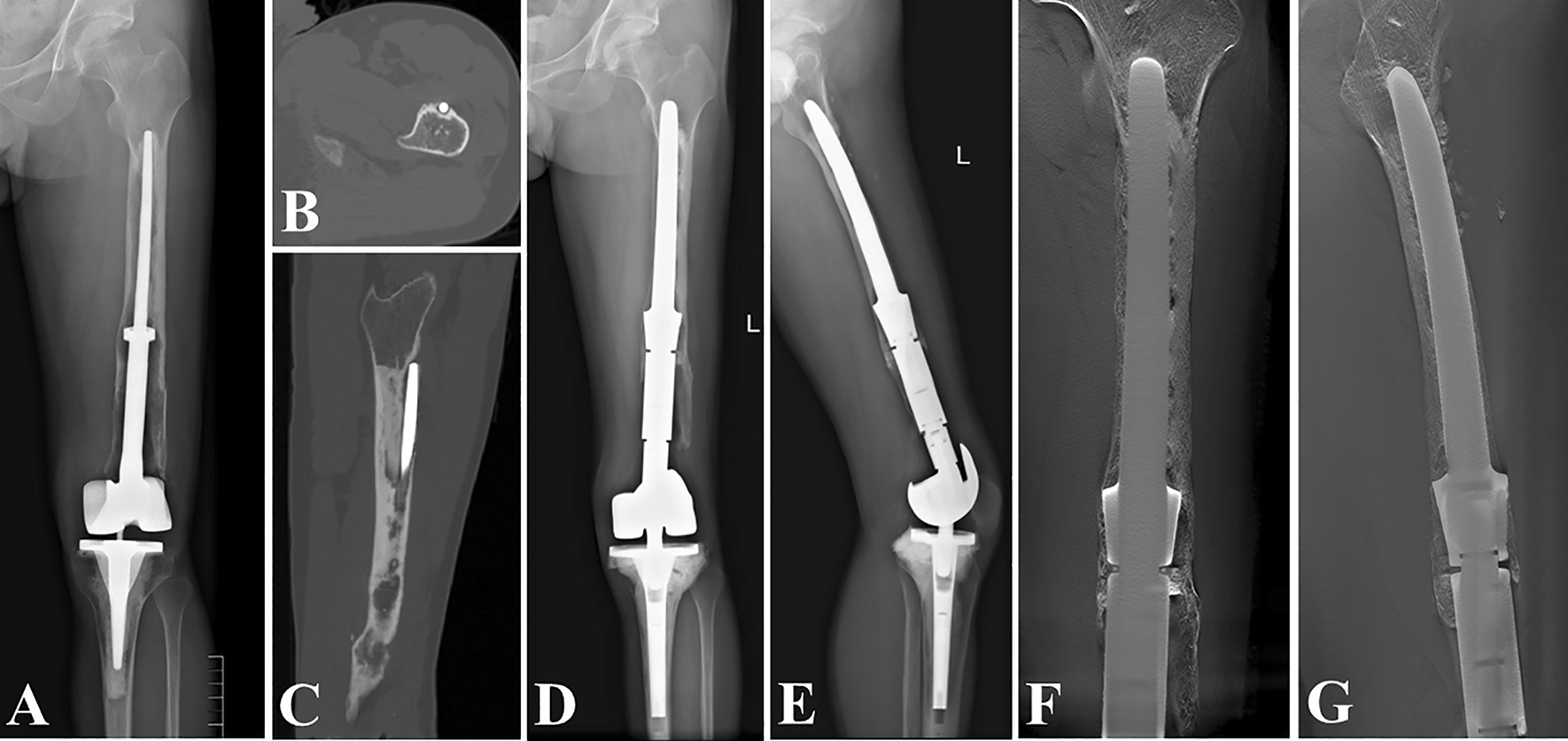
Revision of a Grade II bone loss case with “Long uncemented stem”. **A**–**C** preoperative radiographs; the anteroposterior and lateral radiographs (**D**, **E**), and the digital tomosynthesis images (**F**, **G**) 3 years after revision surgery

**Fig. 5:**
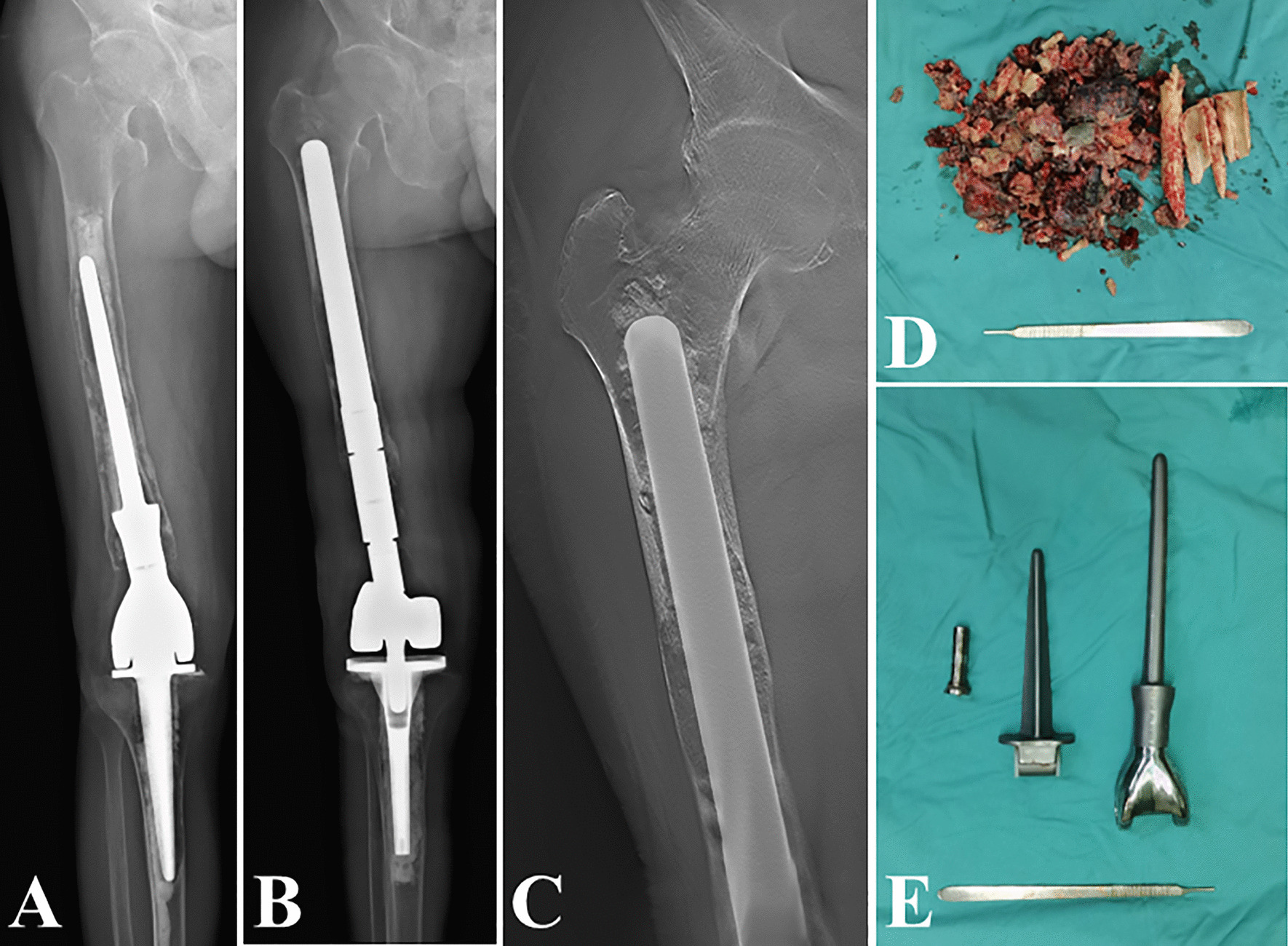
Revision of a Grade III bone loss case with “Ultra-long uncemented stem”. **A**, **B** preoperative radiographs; **C** the digital tomosynthesis image 6 months after revision surgery; **D** the surrounding hyperplastic tissue photograph; **E** the initial cemented endoprosthesis photograph

**Fig. 6:**
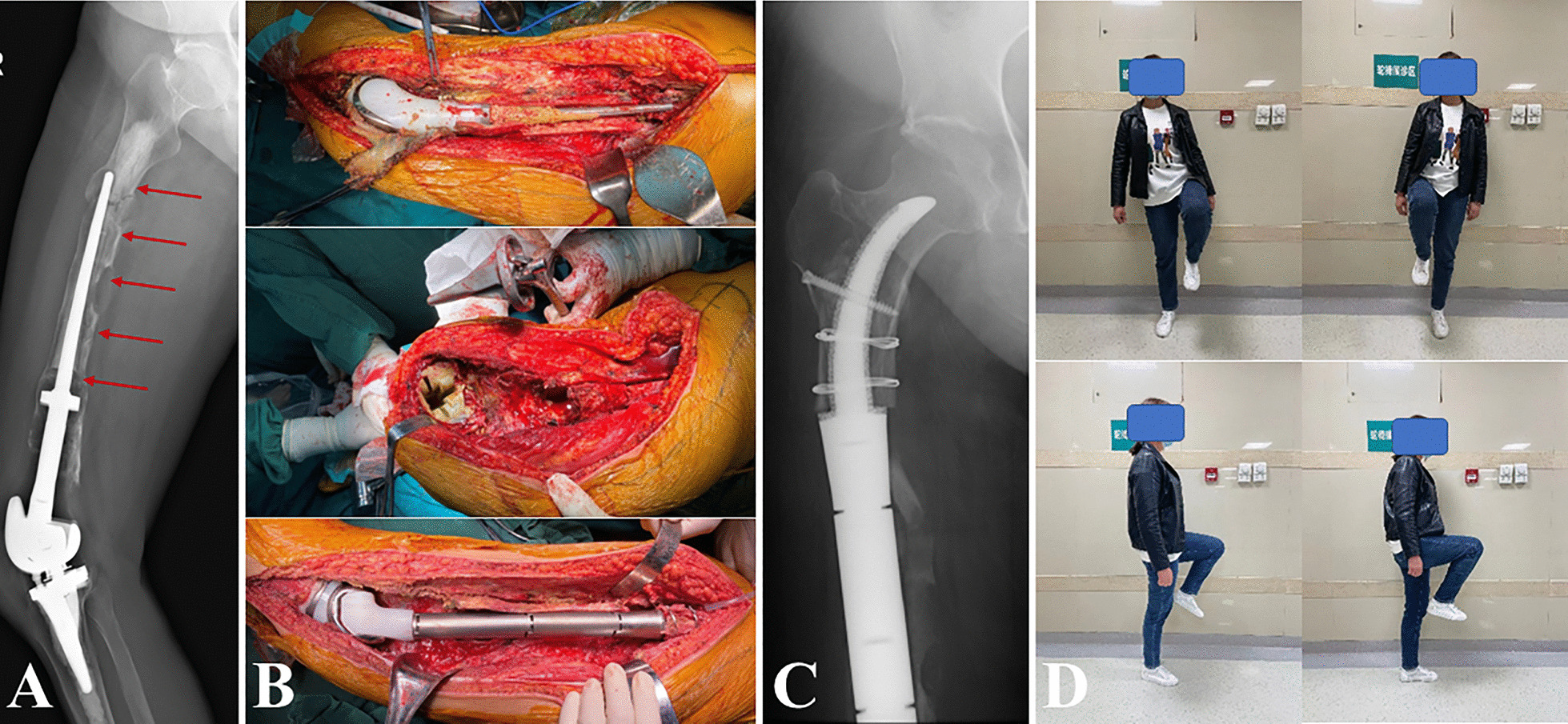
Revision of a Grade IV bone loss case with “Intra-neck curved uncemented stem”. **A** preoperative radiograph; **B** intraoperative photographs; **C** postoperative radiograph 6 months after the revision surgery; **D** function photographs

Following the evaluation and classification of the bone loss, the second step involved reconstructing the 3D computer models and measuring the diameter of the medullary cavity and the anterior curvature of the femur (Fig. 2). The patients’ CT scans data (DICOM format) was collected and imported into Mimics Software (Materialise Corp. Belgium) to build 3D models of the residual femur and initial DFR. Based on the Mimics images, the diameter of the medullary cavity was measured at 1 cm intervals for the revision stem’s diameter design. The anterior curvature of the femur was measured, and the radian of the curved stem was adjusted to match the femur. For the Grade I bone loss case, the “short stem” (the tip of the revision stem did not need to exceed the initial stem) was considered (Fig. 3). For the Grade II bone loss case, the “long stem” (the tip of the revision stem exceeding the perforation 1-2 cm) was considered (Fig. 4). While for the Grade III bone loss case, the “ultra-long stem” (the tip of the revision stem exceeding the initial stem or perforation 6 cm at least) was considered (Fig. 5). Besides the diaphysis curved uncemented stems, an intra-neck curved uncemented stem with a porous interface was designed for the Grade IV bone loss case (Fig. 6).

All revision stems were fabricated by Chunli Co., Ltd. (Tongzhou, Beijing, China). The diaphysis curved uncemented stems were manufactured through the forging method and coated with titanium and hydroxyapatite. The intra-neck curved uncemented stem was manufactured using the electron beam melting technique (ARCAM Q10plus, Mölndal, Sweden), and the printing raw material was Ti-6Al-4 V powder.

### Surgical technique

All surgeries were performed by the same senior surgeon. Under general anesthesia, the original incision was selected to expose the initial DFR. After removing the surrounding hyperplastic tissue, the loosed femoral stem was extracted. The granulation tissue and wear debris were cleaned away. The bone cement in the medullary cavity was taken out as much as possible. After that, the femoral medullary cavity was reamed using flexible reamers. To achieve a precise match between the stem and the medullary cavity, gradient reaming was used according to the gradient diameter of the revision stem. Autologous cancellous bone was grafted around the canal, and then the stem was inserted into the prepared medullary cavity. For patients’ endoprosthetic components needing replacement, the tibial endoprosthesis along with the bone cement were removed. But all the revision tibial endoprosthesis was fixed by the cement technique. During the procedure, the blood vessels and nerves of the popliteal fossa were carefully protected (Table 3).

**Table 3:** The details of bone defect filled or partially restored

Bone loss grades	Case	Peri-stem remaining defect	Bone defect filled	Bone defect partially restored
Grade I	8	1	1	0
Grade II	5	4	2	2
Grade III	3	2	1	1
Grade IV	1	0	0	0

### Postoperative management and follow-up

The operative limb was kept non-weight bearing for 4–6 weeks after revision surgery. Thereafter, patients were encouraged to gradually increase weight-bearing on the affected limb. All patients were followed monthly during the first 3 months, and every 3 months thereafter. The oncologic outcomes and complications were recorded. At the last follow-up, the pain level was evaluated using the VAS method, and the functional outcome was evaluated using the MSTS scoring system. The radiologic evaluation was also performed by comprising anteroposterior and lateral radiographs. In detail, the peri-stem remaining bone defect after insertion of the 3D design stem was evaluated by immediate postoperative radiographs. The change in the bone of the stem bed, such as the filling of postoperatively remaining bone defects, was determined according to the radiologic follow-up.

## Results

The mean period between the primary endoprosthetic reconstruction and the revision surgery was 7.5 years (range, 2 to 17 years). The patient counts with their corresponding Grades of femoral bone loss after aseptic loosening were as follows: Grade I, 8 patients; Grade II, 5 patients; Grade III, 3 patients; and Grade IV, 1 patient.

### Clinical results

Patients were followed for at least 2 years with a mean of 80 months (range, 25 to 126 months), of whom 12 cases were followed for more than 5 years. At the last follow-up, all the patients were alive without local recurrence or distant metastasis. During the follow-up, no revision failure was detected, and no further revision surgery was performed. However, delayed wound healing was observed in two of the 17 patients. Debridement and closure procedures were performed, and the wounds healed after 1 month in both patients. In addition, no other complications were observed, such as infection, local recurrence, and dislocation. The VAS score improved from a median of 5 points (range 3 to 7) preoperatively to 1 point (range 0 to 3) at the last follow-up. The MSTS score improved from a median of 16 points (range 13 to 19) preoperatively to 26 points (range 22 to 28) at the last follow-up. There was no limitation in the range of motion of the knee joint or daily function.

### Radiologic results

**Fig. 7:**
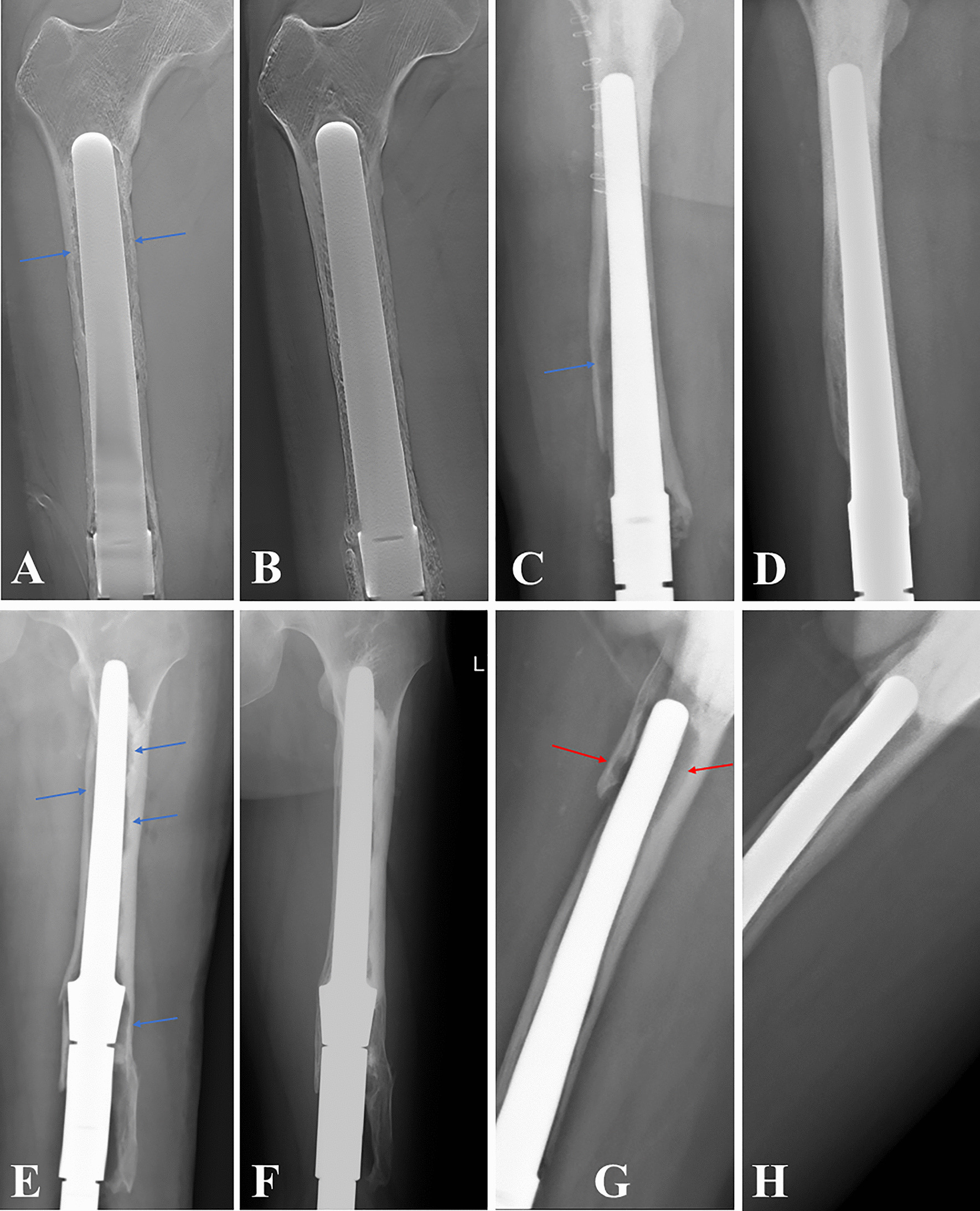
Radiographs demonstrating that the peri-stem remaining defect can be filled or partially restored during the follow-up. The bone defect was filled in the proximal femoral (**A, B**), distal femur (**C, D**), and both proximal and distal femur (**E, F**). The bone defect partially restored (**G, H**)

The postoperative immediate radiographic showed that the revision stem matched the femoral anterior curvature well. The femoral bone defect was completely filled by the 3D design stem in 10 of the 17 cases. In the remaining cases, persistent peri-stem defect was observed. The majority of the defects were located in the proximal femur (Fig. 7). At the last follow-up examination, defects were no longer radiologically visible in 4 of the 7 femurs. Partial restoration of defects could be observed in the remaining 3 femurs. The area of osteolysis was also markedly reduced. Re-aseptic loosening did not occur, and postoperative development of new femoral bone loss was not observed.

## Discussion

Cemented DFR is widely used in clinical practice for the reconstruction of distal femoral bone defects after tumor resection because of its convenience and immediate stability [14]. However, the risk of aseptic loosening is high, especially in patients with long-term follow-up [15]. Revision following aseptic loosening remains challenging even though several methods are available. In the present study, 3D design uncemented stems were used for revision of loosed cemented DER. During a mean follow-up of 80 months, encouraging results were achieved without revision failure. Additionally, satisfactory clinical outcomes were observed in all patients, with significant reduction in pain and improvement in limb function.

Aseptic loosening of the initial DFR often results in host bone absorption and destruction, making a poor bone implantation environment for revision endoprosthesis [5]. Therefore, revision endoprosthesis is prone to postoperative complications and even endoprosthetic failure [14]. According to Bernthal et al. study regarding the revision of loosed DFR by cementing a stem into the existing cement mantle, the long-term survival rate was only 34% for initial revision and 39% for subsequent revision implants [9]. Better results were reported by Mittermayer et al. using an uncemented endoprosthesis for revision in 15 patients, with four of them requiring a further revision procedure [16]. All these results indict the difficulty of creating a stable and durable fixation in revision surgery whether using cemented or uncemented endoprosthesis. But all revision endoprostheses were well fixed in our revision series, and no further revision was performed. In fact, uncemented endoprosthesis relies on the stem press-fitting with the host bone to achieve initial stability and subsequent osseointegration augmented for long-term stability [17]. To achieve precise and stable fixation, all revision stems used in our series were custom-made for each patient on the basis of precise measurement of the residual femoral medullary cavity at 1 cm intervals. Revision stem with gradient diameters combined with gradient reaming enabled press-fit with host femur canal at most of the insert region. And the postoperative immediate radiographic showed that the femoral bone defect was completely filled by the 3D design stem in 10 of the 17 cases. Moreover, persistent peri-stem defects were filled or partially restored in all remaining 7 cases.

In addition, the femur diaphysis has a certain physiological anterior curvature. The curved stem should theoretically be able to better match the medullary cavity compared to the straight stem [18]. However, most commercially available DFR provided a straight femoral stem design [19]. Likewise, all initial femoral stems were also straight in our series, not matching the anterior curvature of the femur. Consequently, the stress is concentrated on the tip of the stem, and the straight stem is at high risk of penetrating the anterior cortex of the femur. To solve the mismatch between the femur and stem, the curvature of the femur was measured, and the radian of the curved stem was adjusted accordingly. The postoperative radiographic results showed the revision stem matching the femoral anterior curvature well. Therefore, excellent results were observed in our revision cases, without implant-related complications, such as aseptic loosening, breakage, or dislocation, which were comparable to the initial reconstruction using uncemented endoprosthesis [20, 21].

Revision of aseptic loosening for patients with severe bone loss is highly surgical demanding. The short residual proximal femur segment cannot be addressed by the traditional stemmed implants. In the present study, severe bone loss was observed in one female patient, with nonsupportive bone at the lesser trochanter. This grade of bone loss is rarely reported in clinical studies. An intra-neck curved uncemented stem with a porous interface was custom-made for this patient through 3D printing technology. During the follow-up of 42 months, the endoprosthesis was in a good position. Compared with total femoral replacement, the hip joint of this patient was preserved, which could lead to better postoperative limb proprioception and function. Additionally, compress osteointegration endoprosthesis is an alternative revision method. However, besides the high risk of complications of this device, the rehabilitation of patients undergoing revision was delayed for 3 months [3].

Bone loss evaluation should be performed in the preoperative period to allow the surgeon to decide which revision strategy is needed [5, 22]. In the present study, the femoral bone loss were evaluated and classified into 4 Grades according to the degree of residual femur deficiency, whether the medullary canal is intact, and whether the medullary canal at the lesser trochanter can be supportive for fixation. For Garde I bone loss, the bone structure around the loosed femoral stem is near normal, and the revision surgery is relatively simple and similar to the initial endoprosthetic replacement. A “short stem” can be considered for revision surgery. For Grade II bone loss, the bone implantation environment was damaged, with the initial femoral stem penetrating the cortical bone. A “long uncemented stem” can be sufficient for revision endoprosthesis fixation. Meanwhile, the longer stem can avoid the position of cortical perforation. For Grade III bone loss, the loosed femoral stem caused extensive cortical thinning with osteolysis, resulting in the bone implantation environment being very poor. Therefore, more proximal femoral bone stock is required for fixation of the revision endoprosthesis, and an ultra-long stem can be considered. And the revision endoprosthesis mainly depends on the fixation of the upper part of the uncemented stem. For Grade IV bone loss, the traditional stemmed endoprosthesis cannot be feasible for revision. 3D printed custom-made porous intra-neck stem is a feasible method.

 There are several limitations of this study. First, it is a single institution experience with the operations carried out by one surgeon. Also, it is a retrospective study with no control or comparison group. Second, the small number of patients in this series does not allow assessment of risk factors that may lead to aseptic loosening.

## Conclusions

3D design uncemented stem matched the femoral anterior curvature well and filled the femoral bone defects at most of the insert region. Moreover, the remaining peri-stem defects can be filled or partially restored during the follow-up. Therefore, the 3D design custom-made uncemented stem created precise, stable, and durable fixation and provided satisfactory clinical outcomes, which is a viable method for revision surgery. In addition, preoperative bone loss evaluation and classification, allowing the surgeon to decide which revision strategy is needed, was a prerequisite for success.

## Data Availability

The datasets used during the current study are available from the corresponding author on reasonable request.

## References

[1] Zhang H-r (2022). Application and development of megaprostheses in limb salvage for bone tumors around the knee joint. Cancer Control.

[2] Tang F, Zhou Y, Min L, Zhang W, Shi R, Luo Y, Duan H, Tu C (2016). Limb-salvage treatment of en-block resected distal femoral tumors with endoprosthesis of all-polyethylene tibial component: a 9-year follow-up study. Onco Targets Ther.

[3] Zimel MN, Farfalli GL, Zindman AM, Riedel ER, Morris CD, Boland PJ, Healey JH. Revision distal femoral arthroplasty with the Compress® prosthesis has a low rate of mechanical failure at 10 years. Clin Orthop Relat Res® 2016;474(2):528–536. 10.1007/s11999-015-4552-y PMC4709327 26394638

[4] Capanna R, Scoccianti G, Frenos F, Vilardi A, Beltrami G, Campanacci DA. What was the survival of megaprostheses in lower limb reconstructions after tumor resections? Clin Orthop Relat Res® 2015;473(3):820–830. 10.1007/s11999-014-3736-1 PMC4317421 24964884

[5] Hou Z-W, Xu M, Zheng K, Yu X-C. Classification and reconstruction of femoral bone defect in the revision of aseptic loosening of distal femoral endoprostheses: a 10-year multicenter retrospective analysis;2022. 10.1186/s12891-022-05885-7 PMC9608886 36303200

[6] Bernthal N, Upfill-Brown A, Burke Z, Ishmael C, Hsiue P, Hori K, Hornicek F, Eckardt J (2019). Long-term follow-up of custom cross-pin fixation of 56 tumour endoprosthesis stems: a single-institution experience. Bone Jt J.

[7] Pala E, Mavrogenis A, Angelini A, Henderson E, Letson GD, Ruggieri P (2013). Cemented versus cementless endoprostheses for lower limb salvage surgery. J Buon.

[8] Pala E, Trovarelli G, Calabrò T, Angelini A, Abati CN, Ruggieri P. Survival of modern knee tumor megaprostheses: failures, functional results, and a comparative statistical analysis. Clin Orthop Relat Res® 2015;473(3):891–899. 10.1007/s11999-014-3699-2 PMC4317408 24874116

[9] Bernthal NM, Hegde V, Zoller SD, Park HY, Ghodasra JH, Johansen D, Eilber F, Eilber FC, Chandhanayingyong C, Eckardt JJ (2018). Long-term outcomes of cement in cement technique for revision endoprosthesis surgery. J Surg Oncol.

[10] Del Buono A, Denaro V, Maffulli N (2012). Genetic susceptibility to aseptic loosening following total hip arthroplasty: a systematic review. Br Med Bull.

[11] Toepfer A, Harrasser N, Petzschner I, Pohlig F, Lenze U, Gerdesmeyer L, Pförringer D, Toepfer M, Beirer M, Crönlein M (2016). Short-to long-term follow-up of total femoral replacement in non-oncologic patients. BMC Musculoskelet Disord.

[12] Min L, Yao K, Lu M, Zhou Y, Wang J, Tang F, Zhang W, Luo Y, Duan H, Tu C (2018). First application of 3D design custom-made uncemented prosthetic stem for distal femoral cemented megaprosthesis revision. Precis Clin Med.

[13] Tsukushi S, Nishida Y, Hirose T, Nakata E, Nakagawa R, Imanishi J, Nakamura T, Nagano A, Tamiya H, Ueda T. Short-term clinical outcomes of Kyocera Modular Limb Salvage System designed cementless stems for the endoprosthetic reconstruction of lower extremities: a Japanese Musculoskeletal Oncology Group multiinstitutional Study. 2022. 10.1186/s12885-022-09873-x PMC9288729 35842696

[14] Geiger EJ, Arnold MT, Hart CM, Greig D, Trikha R, Sekimura T, Eckardt JJ, Bernthal NM. What is the long-term survivorship of primary and revision cemented distal femoral replacements for limb salvage of patients with sarcoma? Clin Orthop Relat Res® 2022;10:1097. 10.1097/CORR.0000000000002333 PMC9928619 35943730

[15] Coathup MJ, Batta V, Pollock RC, Aston WJ, Cannon SR, Skinner JA, Briggs TWR, Unwin PS, Blunn GW (2013). Long-term survival of cemented distal femoral endoprostheses with a hydroxyapatite-coated collar: a histological study and a radiographic follow-up. JBJS.

[16] Mittermayer F, Windhager R, Dominkus M, Krepler P, Schwameis E, Sluga M, Kotz R, Strasser G (2002). Revision of the Kotz type of tumour endoprosthesis for the lower limb. J Bone Jt Surg Br.

[17] Campbell D, Mercer G, Nilsson KG, Callary SA (2010). Case report: cementless stem stabilization after intraoperative fracture: a radiostereometric analysis. Clin Orthop Relat Res.

[18] O’Donnell PW, Griffin AM, Eward WC, Sternheim A, Wunder JS, Ferguson PC (2014). Early follow-up of a custom non-fluted diaphyseal press-fit tumour prosthesis. Int Orthop.

[19] Hu X, Lu M, Wang Y, Wen Y, Tan L, Du G, Zhou Y, Luo Y, Min L, Tu C (2022). Cementless curved endoprosthesis stem for distal femoral reconstruction in a Chinese population: a combined anatomical & biomechanical study. BMC Musculoskelet Disord.

[20] Tsukushi S, Nishida Y, Hirose T, Nakata E, Nakagawa R, Nakamura T, Imanishi J, Nagano A, Tamiya H, Ueda T (2022). Short-term clinical outcomes of Kyocera Modular Limb Salvage System designed cementless stems for the endoprosthetic reconstruction of lower extremities: a Japanese Musculoskeletal Oncology Group multi-institutional study. BMC Cancer.

[21] El Ghoneimy AM, Shehab AM, Farid N (2022). What is the cumulative incidence of revision surgery and what are the complications associated with stemmed cementless nonextendable endoprostheses in patients 18 years or younger with primary bone sarcomas about the knee. Clin Orthop Relat Res.

[22] Barret H, Laumonerie P, Delclaux S, Arboucalot M, Bonnevialle N, Mansat P (2021). Revision total elbow arthroplasty with the semiconstrained Coonrad/Morrey prosthesis: follow-up to 21 years. JBJS.

